# Crystal structure of CobK reveals strand-swapping between Rossmann-fold domains and molecular basis of the reduced precorrin product trap

**DOI:** 10.1038/srep16943

**Published:** 2015-11-30

**Authors:** Shuang Gu, Oleksandr Sushko, Evelyne Deery, Martin J. Warren, Richard W. Pickersgill

**Affiliations:** 1 Chemistry & Biochemistry Department, School of Biological and Chemical Sciences, Queen Mary University of London, Mile End Road, London, E1 4NS, UK; 2 School of Biosciences, University of Kent, Giles Lane, Canterbury, Kent CT2 7NJ, UK

## Abstract

CobK catalyzes the essential reduction of the precorrin ring in the cobalamin biosynthetic pathway. The crystal structure of CobK reveals that the enzyme, despite not having the signature sequence, comprises two Rossmann fold domains which bind coenzyme and substrate respectively. The two parallel β-sheets have swapped their last β-strands giving a novel sheet topology which is an interesting variation on the Rossmann-fold. The trapped ternary complex with coenzyme and product reveals five conserved basic residues that bind the carboxylates of the tetrapyrrole tightly anchoring the product. A loop, disordered in both the apoenzyme and holoenzyme structures, closes around the product further tightening binding. The structure is consistent with a mechanism involving protonation of C18 and *pro-R* hydride transfer from NADPH to C19 of precorrin-6A and reveals the interactions responsible for the specificity of CobK. The almost complete burial of the reduced precorrin product suggests a remarkable form of metabolite channeling where the next enzyme in the biosynthetic pathway triggers product release.

Cobalamin (vitamin B12) is a cofactor for two enzymes, methylmalonyl-CoA mutase and methionine synthase, and is therefore an essential dietary requirement for humans. Around thirty biosynthetic steps are required to manufacture cobalamin in bacteria and archea, making cobalamin biosynthesis one of the most intricate in nature. We are interested in the synthesis of the corrin ring component, cobinamide, from the ubiquitous tetrapyrrole primogenitor uroporphyrinogen III by a series of reactions including eight S-adenosyl-L-methionine-dependent methylations, ring contraction and reduction^1^,^2^. Many of the intermediates in the pathway are labile and have only recently been identified in non-esterified form^3^. In this paper we investigate the structure of the precorrin-6A reductase CobK involved in the aerobic pathway (Fig. 1), there is a homologous enzyme CbiJ involved in the anaerobic pathway^4^ both enzymes catalyse the NADPH-dependent reduction of precorrin-6A^5^ to the dihydro derivative precorrin-6B^6^. Deuterium labelling and NMR spectroscopy demonstrated a hydride from NADPH is transferred to C19 of precorrin-6A before this intermediate is subsequently converted enzymatically into hydrogenobyrinic acid^6^.

The Rossmann fold was first identified in the dinucleotide-binding proteins^7^ and is one of the most prominent folds in the Protein Data Bank^8^. The Rossmann fold binds a mononucleotide and is comprised of a βαβαβ motif. Therefore the fold that binds dinucleotides such as NADPH involves two such mononucleotide binding motifs related by a pseudo two-fold axis which form a six-stranded parallel β-sheet flanked by α-helices. The two specific features of a canonical Rossmann fold are the Gly–X–Gly–X–X–Gly sequence fingerprint (the glycine-rich loop), and a βαβ motif, which contacts the dinucleotide pyrophosphate moiety. However, CobK does not have the pyrophosphate-binding sequence fingerprint that implies the presence of a Rossmann fold which raises the question of how NADPH is bound. A second question to be resolved is how CobK selects precorrin-6A as substrate from the structurally similar intermediates in the pathway from uroporphyrinogen III to hydrogenobyrinic acid (Fig. 1). Further intrigue accrues from the recent observation that CobK traps its product precorrin-6B^3^ and the molecular mechanism that leads to product-trapping and subsequent release demands structural investigation.

## Results

We have determined the structure of apoenzyme, holoenzyme and a ternary complex with NADPH and product giving unprecedented insights into the structure and mechanism of CobK. The description of the structure here will initially be based on the high-resolution structure of the ternary complex, and then differences between the structures of the ternary complex and the other structures will be described (Table 1 gives details of the resolution, data quality, and refinement). CobK unusually comprises two parallel β-sheet domains arranged so the β-strands point towards the centre of the molecule where the NADPH and substrate bind (Fig. 2). The structures reveal how coenzyme and substrate bind to the active centre and explain the specificity of CobK for precorrin-6A as well as elucidating the molecular basis of product-trapping.

## Quality of the structures

The electron density at 1.32 Å resolution is excellent for the ternary complex with clear density for all residues, product and coenzyme with the exception of the nicotinamide of the coenzyme which is less well defined indicating its mobility. The apoenzyme and holoenzyme structures at 3.17 Å and 1.63 Å respectively lack the β2/β3 loop which is disordered and the apoenzyme structure is less well defined overall than the binary and ternary complexes (Table 1). The N- and C-terminal domains have a relatively small contact surface, there is only 304 Å^2^ of solvent accessible surface buried between the two domains in the apoenzyme and only two connections both comprising irregular polypeptide chain; therefore there is considerable opportunity for the domains to move with respect to one another. The presence of coenzyme, which buries 361 Å^2^, limits this movement and the presence of precorrin product provides further rigidity. The clarity of the electron density map of the bound precorrin is remarkable. The precorrin molecule buries 607 Å^2^ including 80 Å^2^ with the coenzyme. The picture therefore is one of increasing rigidity of the structure as first NADPH and then precorrin binds.

## CobK comprises β-strand exchanged dinucleotide binding domains

CobK comprises two domains, a coenzyme binding domain and a substrate-binding domain, both of β/α architecture (Fig. 2a–c). These domains are of approximately equal size, 120 residues, and appear closely similar each with three layer α/β/α sandwich structure. From the N-terminus the polypeptide chain first forms the coenzyme-binding domain, a Rossmann-like fold with five well-defined parallel β-strands (β1 to β5) of order 32145; the C-terminal part of the polypeptide of CobK returns after forming the substrate-binding domain and this irregular polypeptide chain is disulfide-linked to β5 (cysteine 95 to 231, Fig. 2d) and forms a vestigial sixth β-strand of the sheet. It is of course unlikely that the disulfide-bond forms in the bacterial cytoplasm, but a large number of other interactions will hold the polypeptide in place in its absence. The central parallel β-sheet of the coenzyme-binding domain is sandwiched between two layers each comprising two α-helices and one irregular loop. The helices on one side are in the β1/β2-loop (α1) and formed by polypeptide close to the C-terminus of the protein (α8). The irregular loop on this side of the sheet is between β2 and β3. On the other side of the sheet, helices α2 and α3 are in the connections between β3 and β4 and β4 and β5, respectively. The irregular loop on this side is formed just before the polypeptide forms the final helix in the structure, α8 (Fig. 2).

The substrate-binding domain comprises six-parallel strands (β6 to β11) of order 432561 with adjacent α-helices and loops; this topology does not feature in the SCOPe database^9^. However, the domain looks very similar to the coenzyme-binding domain with the central parallel β-sheet sandwiched between two layers each comprising two α-helices and one irregular loop. If the first β-strand is ignored for the purposes of comparison then the strand order becomes the more familiar 32145 which is the order of the N-terminal domain and is a core component of many β/α protein superfamilies. In essence, the two parallel β-sheet domains have exchanged their last β-strands giving a twist on a familiar structural theme (Fig. 2a–c). The loops β6/β7, β7/β8, β8/β9 carry the two α-helices (α4 and α5) and irregular loop on one side of the central sheet. The polypeptide extending from the coenzyme-binding domain to form the substrate-binding domain forms the long irregular loop on the other side of the sheet (before β6), supplemented by the helices α6 and α7 in the β9/β10 and β10/ β11 loops, respectively.

It is unusual for both the coenzyme-binding and substrate-binding domains of an enzyme to be formed from parallel β-strand dinucleotide binding-like folds; their relative orientation in CobK means that the strands of the two parallel β-sheets point towards the centre of the molecule. The loops C-terminal to the parallel β-strands form the coenzyme and substrate binding sites. The striking similarity in the structure of the two domains can be seen when they are superimposed ignoring the final irregular chain/β-stand (Fig. 2c). The central five strands can be superimposed with 89 equivalent residues having rmsd of 3.0 Å (Z-score 8.8); but there is no detectable sequence similarity between the two domains. In fact, it is notable that although CobK comprises two Rossmann-fold domains, neither has the characteristic signature sequence.

The DALI server^10^ finds both domains of CobK to be similar to NAD(P)-binding domains when the domains are individually used as search molecules. The highest structural similarity detected is between the NADPH-binding domain of CobK and that of L-lysine dehydrogenase (PDB code: 2Z2V) where 114 residues of the NAD(P)H domains align with rmsd 2.6 Å (Z score 12.2). The sequence identity is 14%. A plausible explanation of how the architecture of CobK arose is that the DNA encoding the NADPH-binding domain of CobK underwent a duplication and as the sequences of the two domains diverged the final β-stands exchanged. The originally identical sequences of the two domains would facilitate this exchange. This would not need unusually lengthy connections between the penultimate and final β-strands, but the residues in the connections should not have high helix-forming propensity for the exchange to occur as they must bridge to the other domain.

## The coenzyme-binding domain lacks any signature nucleotide-binding sequence

Coenzyme binds between the two domains making contacts with the N-terminal, coenzyme-binding domain (Fig. 3a). The sides of the adenosine–binding pocket is formed on one side by the β3/α2 loop and on the other by the β4/α3 loop α3. The floor of the pocket is provided by the surface of the parallel β-strands β1, β2, β3, β4 (Fig. 3a). In the holoenzyme the β2/β3 is disordered and the nicotinamide of the NADPH is not clearly defined. Large hydrophobic residues forming the adenosine-binding site are F50 and M79 from the β3/α2 loop and the N-terminus of helix α3, respectively. The 2’-phosphate binding site of NADPH is formed by main-chain amides of residues Phe50, Gly51 and Gly52 of the glycine-rich β3/α2-loop and by the NH2 of Asn82 from helix α2. The pyrophosphate-binding region of the coenzyme-binding domain has no glycine-rich fingerprint typical of NAD(P)-binding Rossmann folds. Contacts between NADPH and enzyme are presented in Supplementary Figure S1 and Supplementary Table S1. There are glycine residues in the polypeptide chain preceding α2 and α3 but no typical fingerprint and as a consequence unlike the typical situation in alcohol dehydrogenase where both the A and N phosphates are close to the N-terminal end of the pyrophosphate-binding helix (and helix-dipole), only the A phosphate is close to the N-terminal end of α2 and makes hydrogen-bonds the helix amides. The A phosphate group is slightly off-axis and not experiencing the full beneficial effect of the helix-dipole. Although the density is good for adenosine moiety the density falls away rapidly after the nicotinamide phosphate because the N phosphate, nicotinamide ribose and nicotinamide make few contacts with the enzyme and are not in a fixed conformation in the holoenzyme.

## The ternary complex of CobK with NADPH and reduced precorrin product

Comparing the structure of the ternary complex with the holoenzyme reveals the ordering of the β2/ β3 loop, residues 33 to 40, across the substrate binding cleft (Fig. 3a). The propionate group on the C-ring of the tetrapyrrole appears to be important in closing this loop (an inventory of interactions involving the loop is presented in Supplementary Table S2). The propionate makes hydrogen-bonds to the main-chain amides of Ala32 and Gly33. The neighbouring acetate locks the C-ring in place by making two salt bridges to Arg173. Meanwhile, Arg34 of the loop is brought into position to hydrogen-bond to the N phosphate and ribose ring oxygen of the NADPH (Fig. 3b). The hydroxyl of Thr35 makes an additional hydrogen–bond to the acetate of the C-ring. These polar interactions along with numerous hydrophobic ones lock the loop over the bound product. Note that it is the binding of product that promotes loop closure even though some of the contacts are to the coenzyme. A complete list of interactions between CobK and precorrin is given in Table 2 and illustrated in Supplementary Figure S2.

The electron density of the ternary complex clearly reveals that it is product that is bound rather than substrate as both C18 and C19 are *sp*^3^ hybridized (Fig. 3c shows the clarity of the map). The product bound to CobK has been further modified by methylation of C5, this is a modification that can be accommodated within the active centre of the enzyme and points to the lability of the intermediate through the crystallization and data collection steps. The origin of this methyl-group is unclear. Four loops from the substrate-binding domain contribute to the substrate-binding site: β7/α5, β8/β9, β9/α6 and β10/α7. Three loops from the coenzyme-binding domain: β1/α1, β2/β3 and β4/α2 also contribute to the binding-site. The extensive network of interactions that bind the precorrin to the enzyme include five conserved basic residues (Fig. 3d). Sequence conservation is shown in Supplementary Figure S3. At the active centre of CobK the coenzyme and product are held with geometry and relative orientation consistent with the hydride having been transferred from C4 of the nicotinamide to C19 of the precorrin-6B product (Fig. 4a).

## *In silico* docking of substrate and product

Using the ternary complex with precorrin removed, both substrate precorrin-6A and product precorrin-6B dock into the active centre in essentially the same orientation as the experimentally determined structure. Substrate and product also successfully dock correctly into the holoenzyme structure provided the nicotinamide of the coenzyme is modelled in the same conformation as seen in the ternary complex (Fig. 4b). The success in docking is presumably because of the extensive interactions that bind the substrate and product to the enzyme and comparison of the bound substrate and product reveal the change in conformation of the precorrin ring driven by the *sp*^2^ to *sp*^*3*^ transformation of C18 and C19 (Fig. 4c).

## Mechanism

Deuterium labelling experiments have shown that the *pro-R* hydrogen is transferred from C4N of the nicotinamide of NADPH to C19 of precorrin-6A. In the trapped product complex, the C4N of the pyridine residue of NADPH is 2.8 Å of C19 of the D-ring of the corrin macrocycle in agreement with the previously reported stereochemistry of hydride transfer (Fig. 4a). The geometry of the product is such that protonation of C18 occurs from the other side of the macrocycle, but in the crystal structure there is no residue positioned to protonate C18. Protonation of C18 is anticipated to occur before hydride transfer to C19 because the pyrrole ring is electron rich and therefore more likely to stabilize a carbocation intermediate. The face of the macrocycle to which hydride is transferred is shielded from water by the nicotinamide which packs against conserved His73. There is bound water close to C18 on correct face of the macrocycle (Fig. 4a) and it is plausible that this is the delivery path for the proton, the alternative is that substrate assists the protonation step. The product is almost entirely inaccessible to solvent in this time and space averaged structure and only the proprionate of the B-ring can be seen emerging from the substrate-binding pocket (Fig. 4d). The NADPH has greater solvent exposure which can be seen when the molecule is turned around (Fig. 4d).

## Discussion

**Figure 1:**
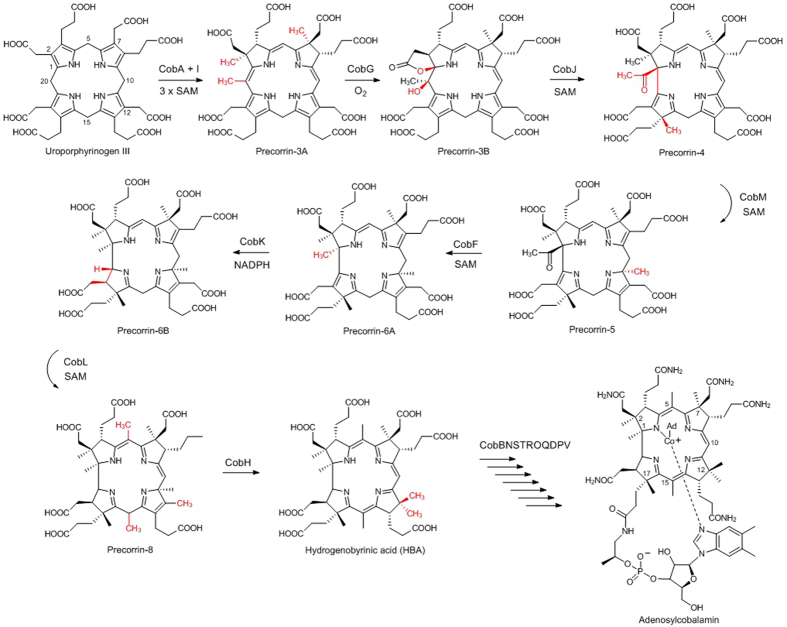
The reaction catalyzed by the precorrin reductase CobK in the context of the eight methyltransferase reactions leading from uroporphyrinogen III to hydrogenobyrinic acid which is subsequently converted to cobalamin (vitamin B12). Precorrin-6A is transformed into precorrin-6B the substrate for the methyltransferase CobL in a reaction dependent on the coenzyme NADPH.

**Figure 2:**
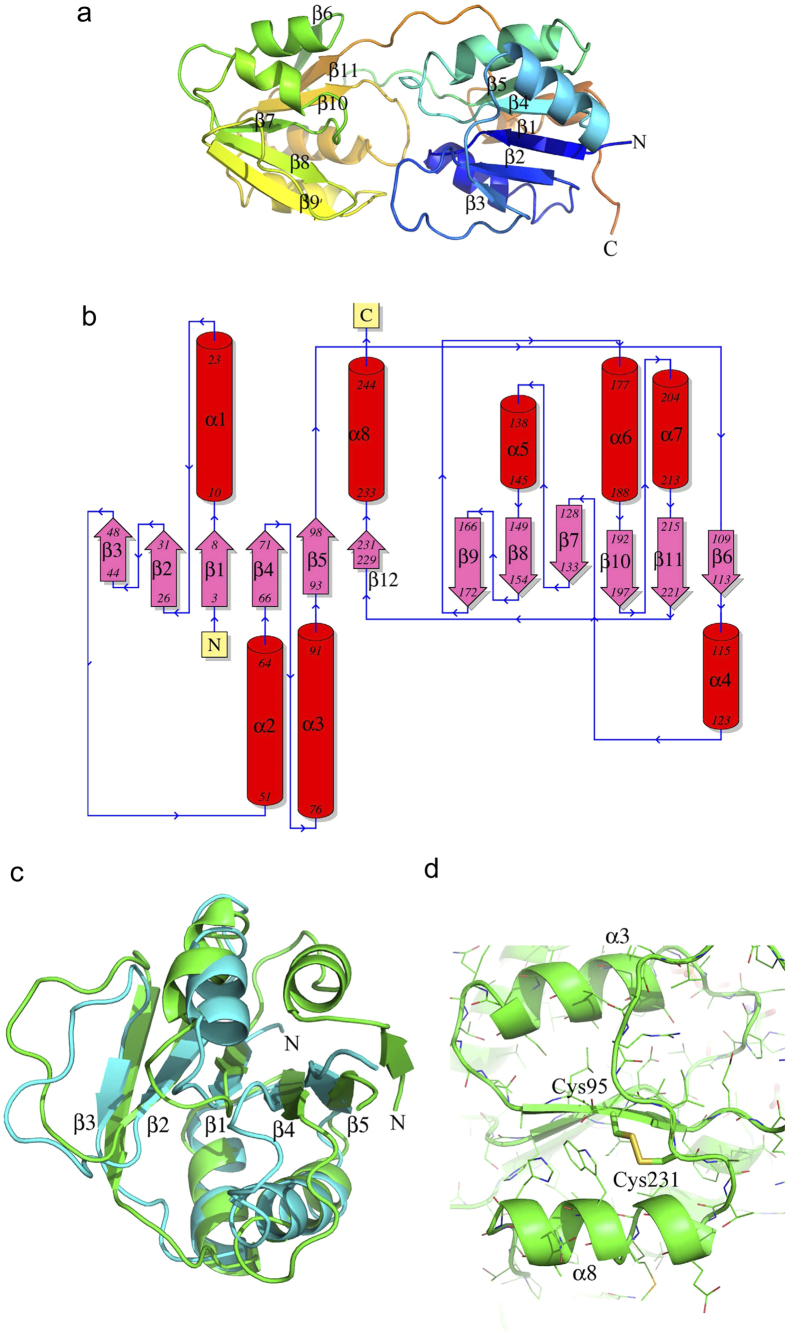
The crystal structure of the precorrin reductase CobK. (**a**) Cartoon structure of CobK reveals two Rossmann-like folds facing each other across the NADPH and substrate binding cleft, the polypeptide chain is coloured from blue to orange from N- to C-terminus. The polypeptide chain crosses from the N-terminal domain to form the C-terminal before returning to complete the N-terminal domain. In essence, the final β-strands are swapped between domains. (**b**) The topology of CobK reveals the strand order in the two Rossmann-like fold domains. (**c**) Superimposition of the two domains (N- and C-terminal in cyan and green, respectively) reveals their topological similarity once the swapped β-stands are excluded (strand order 32145). (**d**) The polypeptide chain returning to the N-terminal domain forms a disulfide (Cys95–Cys231) with β5 and then forms the final helix, α8. This and the other figures of the structures were made using PyMOL, unless otherwise indicated.

**Figure 3:**
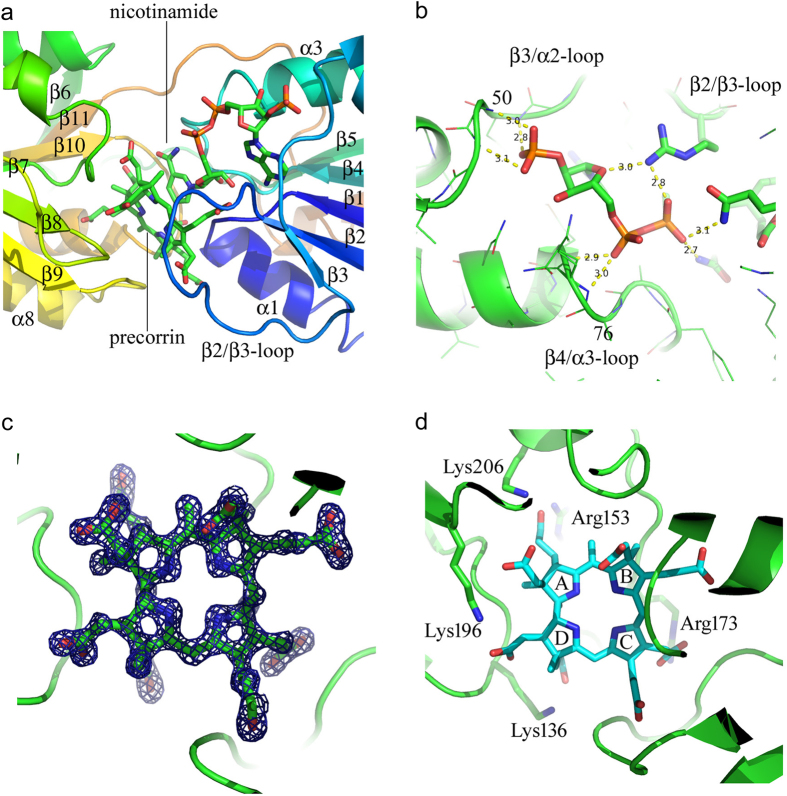
The crystal structure of the ternary complex. (**a**) The binding sites of coenzyme NADPH and precorrin product relative to the domain architecture of CobK with β2/β3 loop (residues 33 to 40) that becomes ordered on product-binding indicated. (**b**) In the holoenzyme structure the nicotinamide phosphate and atoms to the nicotinamide side of the coenzyme are not seen, but in the ternary complex Arg34 makes hydrogen bonds to the nicotinamide phosphate (NP) and ribose ring oxygen and the coenzyme is much better defined. Adenosine binds on the surface of the β-sheet with the 2’-phosphate binding amides of residues 50, 51 and 52. Phe50 and Met79 contribute to the hydrophobic pocket binding the adenosine. The adenosine phosphate (AP) binds main chain amides of residues 78 and 79 at the N-terminal end of helix α3, but beyond the nicotinamide phosphate (NP) the coenzyme conformation is poorly defined in the holoenzyme structure and is presumed to be mobile. (**c**) Electron density corresponding to the precorrin product showing great clarity and enabling the contacts between enzyme and product to be confidently assigned. This σ_A_-weighted 2Fobs-Fcalc map is contoured at 1.5σ. (**d**) Charged residues that interact with the precorrin product. These are conserved basic residues belonging to the C-terminal domain. The NADPH has been removed from the top of the product in this panel to reveal the interactions with charged residues. Note that the precorrin orientation is the same in Figures: 1; 3c; 3d; 4b and 4c with the rings labelled in Fig. 3d. LIGPLOT figures of the ligand interactions and the sequence alignment are shown as supplementary Figures S1, S2 and S3, respectively.

**Figure 4:**
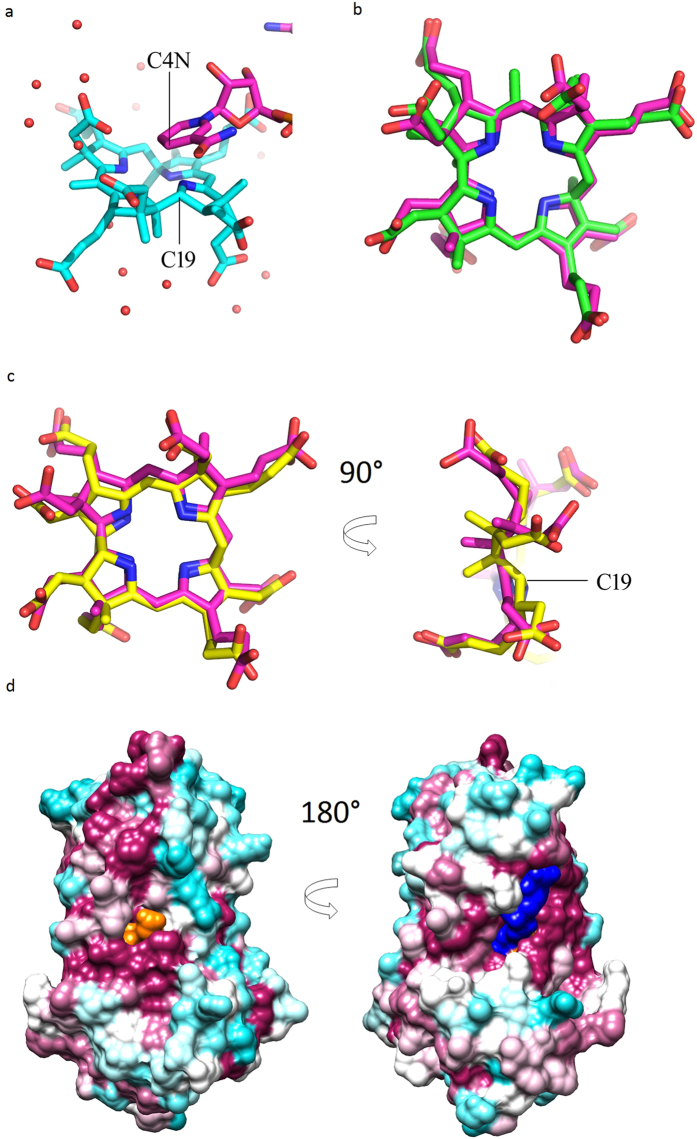
Product binding in detail. (**a**) NADPH and precorrin drawn in stick representation showing their relative orientation within the active centre of CobK. The geometry and separation of nicotinamide C4N and precorrin C19, 2.8 Å, is consistent with hydride transfer from C4N to C19. The carbons of the nicotinamide and precorrin are shown in magenta and cyan respectively. Oxygen atoms are red and nitrogen atoms blue; the small red spheres are water molecules shown as the proton may be provided by water to C18. (**b**) *In silico* the product precorrin-6B (magenta carbons) docks is a closely similar orientation to the bound product seen in the experimental structure (green carbons). (**c**) *In silico* the substrate precorrin-6A (yellow carbons) also docks into the active centre of CobK in similar orientation to that observed experimentally and clearly shows the changes in conformation that accompanies reduction of the C18-C19 bond. (**d**) Surface representation of the ternary complex. CobK surface coloured according to conservation in magenta (highly conserved) to cyan (poorly conserved), precorrin surface in orange, and NADPH surface in blue. The figure highlights the almost complete burial of the precorrin, this is the only surface of the precorrin visible in any orientation of the molecule and only the proprionate of ring-B can be seen protruding close to the conserved sequences that bind the substrate and product. This panel was produced using Chimera^20^.

At the beginning of this work it was not clear if CobK had a Rossmann-like fold; the structure reveals that it has not one but two Rossmann-like folds with their parallel β-sheets pointing towards the centre of the molecule where coenzyme and substrate bind. The swapping of the last β-strand of the N-terminal domain and vestigial disulphide-bonded β-strand of the C-terminal domain has not been seen before. The CobK apoenzyme has few contacts between the coenzyme and substrate binding domains; the structure becomes increasingly rigid as first coenzyme binds and then product and this is reflected in the resolution to which the respective crystals diffracted. There are subtle changes on binding both coenzyme and substrate, but ordering of the β2/β3 loop is a clear and distinctive change that further hides product from water (Fig. 4d). The most plausible mechanism for rate enhancement by CobK involves protonation of C18 of the precorrin by a water molecule and *pro-R* hydride transfer from NADPH to C19 (Fig. 4a). It is clear from the product complex why precorrin-6A is the preferred substrate for CobK. There is no room to fit a non-contracted macrocycle or one with the gamma-lactone group on C1 of precorrin-3B through precorrin-5 into the active centre. After decarboxylation of C12 by the next enzyme, CobL, the interaction of the C12 carboxylate and Arg173 is lost, so precorrin-8 and later intermediates will not bind so tightly and may not cause loop closure (Fig. 1).

The key question now to be answered is how CobL, the next enzyme in the biosynthetic pathway, releases product from CobK. The current structure is an important stepping stone to answer this key question.

## Methods

### Protein production and crystallization

CobK was cloned from *Rhodobacter capsulatus* SB1003 and purified as described previously^3^. The purified protein was the apoenzyme, holoenzyme was formed by the addition of 5 mM NADPH, and ternary complex by addition of 5 mM NADPH and excess isolated precorrin produced from a plasmid encoding CobA-I-G-J-M-F-K^3^. Crystals of CobK apoenzyme, holoenzyme and ternary complex were grown by hanging drop vapour diffusion using protein at 20 mg/ml and drops of 2 μl protein and 2 μl reservoir comprising 18% PEG3350, 0.2 M ammonium chloride. Selenomethionine labelled protein crystals of the ternary complex were grown using similar conditions to the apoenzyme and holoenzyme.

### Structure solution

Protein phases for the apoenzyme were determined using single-wavelength anomalous dispersion from an osmium derivative made by soaking a crystal in reservoir augmented with 20% glycerol as cryopotectant and 10 mM potassium osmate for 90 minutes. The CobK ternary complex was solved independently using single-wavelength anomalous dispersion from selenomethione labelled protein. X-ray diffraction data were collected using the Proxima 1 beamline at Soleil and the data were processed using XDS^11^. Protein phases were determined using ShelxCDE^12^, initial models were built using Coot^13^ and refined using Buccaneer^14^. The holoenzyme structure was solved using molecular replacement, Phaser MR^15^ from the CCP4 suite^16^ using the native structure. LIGPLOT^17^ produced representations of the contacts between CobK and ligands. Data collection and refinement statistics are presented in Table 1.

### Molecular modelling

Intramolecular restraints for the precorrin ligands were generated using PRODRG^18^. Autodock Vina^19^ was then used to dock precorrin-6A and precorrin-6B into the structure of the apo- and holo-enzymes.

## Additional Information

**Accession Codes**: Coordinates and structure factors are deposited in the protein databank with codes 5C4R, 5C4N and 4X7G for the apoenzyme, holoenzyme and ternary complex respectively.

**How to cite this article**: Gu, S. *et al*. Crystal structure of CobK reveals strand-swapping between Rossmann-fold domains and molecular basis of the reduced precorrin product trap. *Sci. Rep*. **5**, 16943; doi: 10.1038/srep16943 (2015).

## Supplementary Material

Supplementary Information

## Figures and Tables

**Table 1:** Crystallographic statistics.

	Apoenzyme	Holoenzyme	Ternary Complex
Data collection statistics			
Space group	P 2_1_ 2_1_ 2_1_	P 2_1_ 2_1_ 2_1_	P 2_1_ 2_1_ 2_1_
Cell edges (Å)	33.9, 75.4, 116.6	33.9, 73.1, 103.2	53.1, 60.3, 76.2
Molecules per asymmetric unit	1	1	1
Wavelength (Å)	1.13956	0.97857	0.97857
Resolution (Å)	46.11–3.17	42.17–1.63	47.3–1.32
High resolution shell (Å)	(3.34–3.17)	(1.72–1.63)	(1.39–1.32)
Number of observations	29900	238973	420749
Number of unique reflections	16610	32962	58303
Multiplicity	1.8 (1.7)	7.2 (7.4)	7.2 (7.1)
Rmerge†	0.138(0.596)	0.062 (0.546)	0.031 (0.078)
Rpim‡	0.138 (0.562)	0.025 (0.214)	0.026 (0.367)
Mean I/σ(I)	5.5.(2.6)	19.4(4.4)	14.2(2.6)
CC_1/2_	0.984 (0.679)	0.999 (0.870)	0.999 (0.717)
Wilson B factor (Å^2^)	51.3	22.5	16.2
Completeness (%)	98 (97)	100 (100)	100 (100)
Refinement statistics			
Resolution	63.01–3.17	42.7–1.63	47.29–1.32
Number of reflections	4968	31248	54587
R-factor/R-free±	0.178/0.227	0.184/0.214	0.170/0.192
Number of atoms (protein/ligand/water)	1796/1/0	1789/47/259	1872/113/190
rmsd bond length	0.0112	0.0216	0.0289
rmsd bond angle	1.61	2.32	2.84
Ramachandran plot			
Residues in most favoured regions (%)	93.4	97.1	97.5
Outliers (%)	0	0	0

^†^R_merge_ = Σ_hkl_ Σ_i_ |I_i_–<I> |/−Σ_hkl_ ΣI_i_, where I_i_ is the intensity of the i^th^ observation, <I> is the mean intensity of the reflection and the summations extend over all unique reflections (hkl) and all equivalents (i), respectively.

^‡^Rpim is a measure of the quality of the data after averaging the multiple measurements and R_pim_ = Σ_hkl_ [n/(n-1)]^1/2^ Σ_i_ |I_i_(hkl)–<I(hkl) >|/Σ_hkl_ Σ_i_ I_i_(hkl), where n is the multiplicity, other variables as defined for R_merge_^21^.

^±^R-factor = Σ_hkl_ |F_o_–F_c_ |/Σ_hkl_ F_o_, where F_o_ and F_c_ represent the observed and calculated structure factors, respectively. The R-Factor is calculated using 95% of the data included in refinement and R-free the 5% excluded. The values presented in this Table are from AIMLESS^22^, PHENIX.REFINE^23^ and PROCHECK^24^ from the CCP4 suite.

**Table 2:** Hydrogen bonds between holoenzyme and precorrin-6B.

Pyrrole ring	Carboxylate	Hydrogen bonds
A	Acetate	(O28) Hydrogen bonds to main chain amide of Lys196 and carbonyl of Ala133 via bound water molecule(O29) Hydrogen bonds to NZ Lys206 and carbonyl of Lys196 via bound water molecule
	Propionate	(O33) Hydrogen bonds to NH1 Arg153, NZ of Lys206 and bound water (water also hydrogen bonds to OD2 Asp181 and NH Lysine 206)(O34) Hydrogen bonds to both NE Arg153
B	Acetate	(O39) Hydrogen bonds to main chain amide of Thr11 and bound water(O40) No clear hydrogen bonding
	Propionate	(O44) Hydrogen bonds to OG1 of Thr11(O45) Hydrogen bonds to OG of Ser14 and bound water
C	Acetate	(O22) Hydrogen bonds to NH2 of Arg173 and OG1 of Thr35(O23) Hydrogen bonds to NE of Arg173
	Propionate	(O51) Hydrogen bonds main chain amides Ala32 and Arg34.(O52) Hydrogen bonds main chain amide of Ala32 and O3’ of nicotinamide ribose ring NADPH
D	Propionate	(O58) Hydrogen bonds to NZ Lys136 and water(O59) Main chain amide of Val55 and water
	Acetate	(O62) Hydrogen bonds to NZ of Lys196, NH2 of Gln137 and a bound water(O63) Hydrogen bonds to main chain amides of Lys136 and Gln137

Hydrogen-bonds involving conserved basic residues are underlined.
