# Supplementary material for: Crystal structure of CobK reveals strand-swapping between Rossmann-fold domains and molecular basis of the reduced precorrin product trap
Source: Sci Rep. 2015 Nov 30;5:16943. doi: 10.1038/srep16943 (PMC4663756; doi:10.1038/srep16943)
Supplement: Supplementary Information [file srep16943-s1.pdf]

Crystal structure of CobK reveals strand-swapping between Rossmann-fold domains and molecular basis of the reduced precorrin product trap

Shuang Gu, Oleksandr Sushko, Evelyne Deery, Martin J. Warren, Richard W. Pickersgill

Table S1. Hydrogen-bonds between CobK and $NADP^+$ in the holoenzyme and ternary complex

$NADP^+$	Holoenzyme	Ternary complex
Ribose 2'-phosphate OX1	NH Gly51	NH Gly51
Ribose 2'-phosphate OX2	NH Gly52 and ND2 Asn82	NH Gly52 and ND2 Asn82
Ribose 2'-phosphate OX3	NH Phe50	NH Phe50
Adenosine N6A	O Gly8 and O Ser30	O Ser30 and O3D NADPH (3'-hydroxyl)
O3B		ND2 Gln78 and O3X NADPH
O1N		NH2 Arg34
N1A		SerOG
Adenosine phosphate O2A	NH Ala77, NH Gln78 and NH Met79	NH Ala77, NH Gln78 and NH Met79
Nicotinamide phosphate O2N	-	ND2 Gln137 and N7N NADPH
O1N	-	NE and NH2 of Arg34
O2D	-	NH His73 and O Gly8
O3D	-	O Gly8, N6A and N7A NADPH
N7N	-	O2N
O7N	-	NZ Lys196

Table S2. Hydrogen-bonds involving the loop ordered in the ternary complex

Gly33	N	PC O51
Arg34	N	PC O51
Arg34	NH1	O1N and O4B
Thr35	OG1	PC O22
Gly36	N	Asp156 OD2
Ala37	O	Arg173 NH2
Val39	N	PC O23
Val39	O	Tyr31 OH

Figure S1. NADP$^+$ contacts in the ternary complex (LIGPLOT)

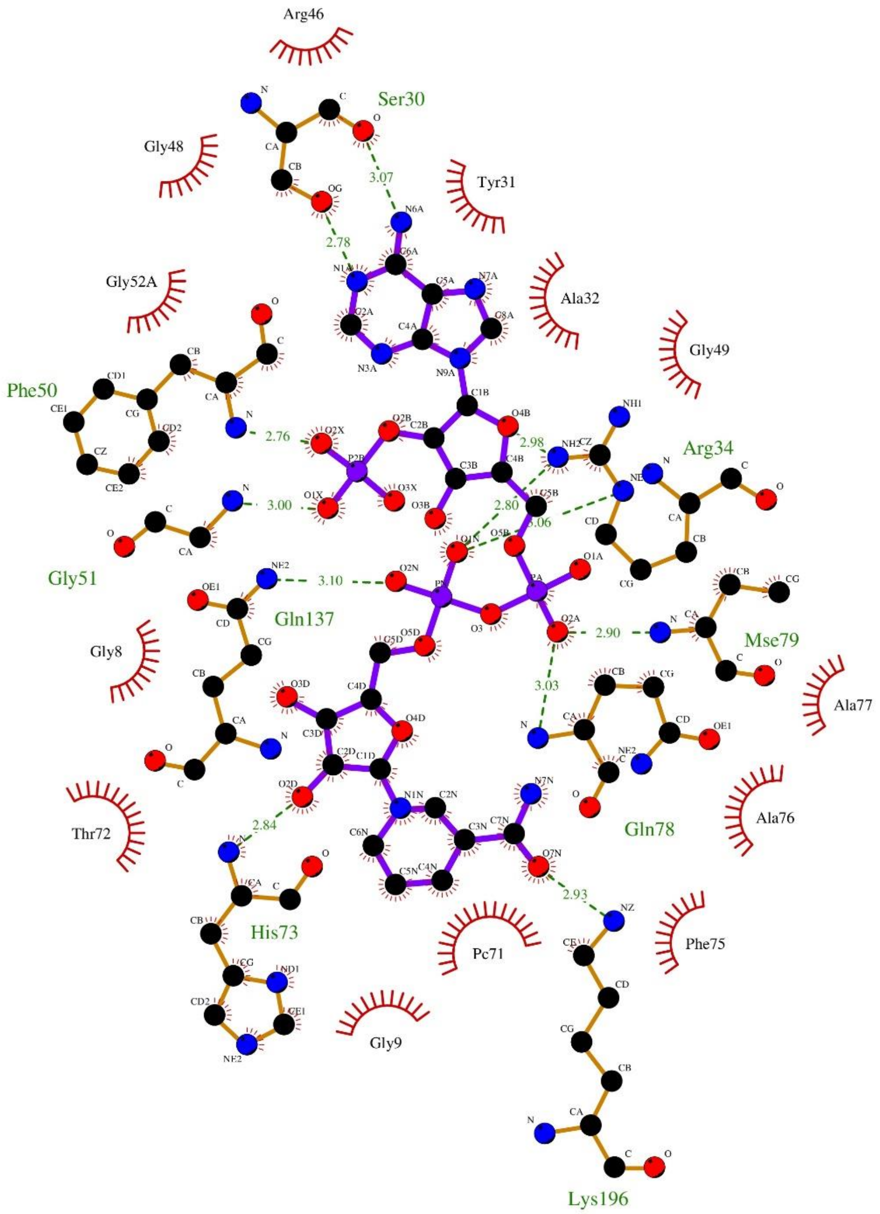

.

Figure S2. Precorrin contacts in the ternary complex (LIGPLOT)

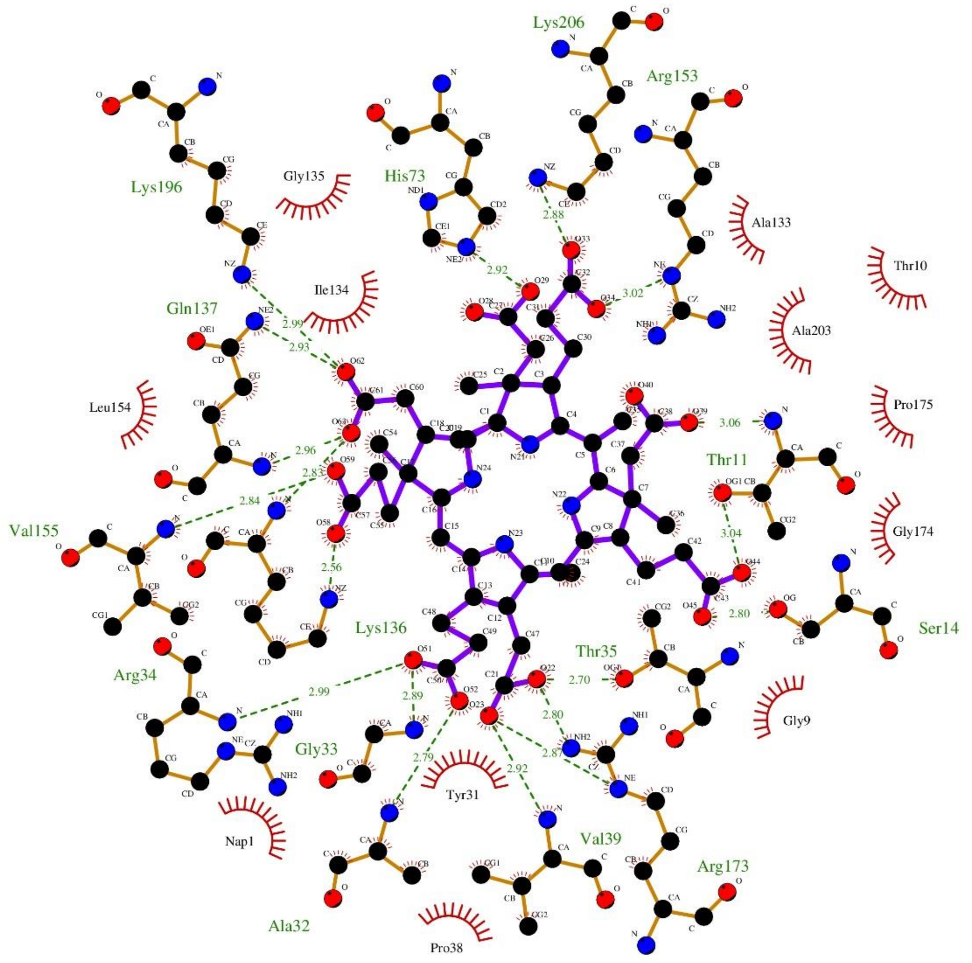

Figure S3. Sequence conservation in CobK

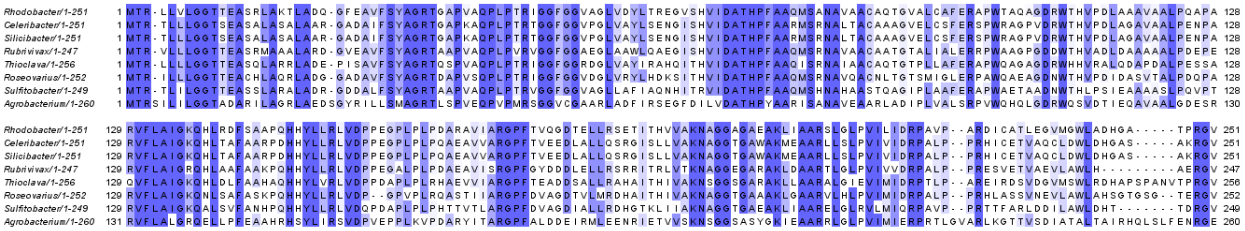

The aligned sequences are from: *Rhodobacter capsulatus, Celeribacter indicus, Silicibacter sp. TrichCH4B, Roseovarius sp. BRH_c41, Rubrivivax benzoatilyticus, Thioclava sp., Sulfitobacter pontiacus, Agrobacterium sp.* The intensity of the shading indicates the level of conservation. The histidine which is a platform for the nicotinamide and the five basic residues binding the substrate and product are conserved along with several other substrate and coenzyme binding residues.
